# A high protein low glycemic index diet has no adverse effect on blood pressure in pregnant women with overweight or obesity: a secondary data analysis of a randomized clinical trial

**DOI:** 10.3389/fnut.2023.1289395

**Published:** 2023-11-21

**Authors:** Elisabeth A. Larson, Faidon Magkos, Helle Zingenberg, Jens Svare, Arne Astrup, Nina R. W. Geiker

**Affiliations:** ^1^Division of Nutritional Sciences, Cornell University, Ithaca, NY, United States; ^2^Department of Nutrition, Exercise and Sports, University of Copenhagen, Frederiksberg, Denmark; ^3^Department of Gynecology and Obstetrics, Copenhagen University Hospital Herlev-Gentofte, Herlev, Denmark; ^4^Department of Obesity and Nutritional Sciences, Novo Nordisk Foundation, Hellerup, Denmark; ^5^Centre for Childhood Health, Copenhagen, Denmark

**Keywords:** animal protein, blood pressure, obesity, pregnancy, hypertension

## Abstract

**Objectives:**

The objective of this analysis was to evaluate the effect of a diet rich in animal protein and low in glycemic index on blood pressure during pregnancy.

**Design:**

This *post hoc*, secondary data analysis of a randomized controlled trial, evaluated blood pressure in pregnant participants who were randomized either to an *ad libitum* diet with high protein and low glycemic index, rich in dairy and seafood, or an *ad libitum* control diet according to national recommendations.

**Setting:**

The study occurred in pregnant women in Copenhagen, Denmark.

**Sample:**

A total of 279 pregnant females with overweight or obesity were enrolled.

**Methods and outcome measure:**

Blood pressure was measured at 5 timepoints during pregnancy from gestational week 15 through week 36, and blood pressure between groups was compared.

**Results:**

There were no differences between diet arms in systolic or diastolic blood pressure over time. There were also no differences in most blood-pressure-related pregnancy complications, including the prevalence of premature birth, preeclampsia, or hypertension, but the frequency of total cesarean sections was lower in the active than the control group (16 out of 104 vs. 30 out of 104) (*p* = 0.02).

**Conclusion:**

Increased animal protein intake was not associated with changes in blood pressure in pregnant women with overweight or obesity.

**Clinical trial registration:**

[ClinicalTrials.gov], identifier [NCT01894139].

## Introduction

During pregnancy, women are confronted with an onslaught of new and sometimes conflicting information about how best to maintain health through this period of metabolic adaptations (1). Differing recommendations complexify prenatal nutritional decision making; and leave questions both for mothers and healthcare providers about how best to support a healthy pregnancy through diet. Some healthcare providers like doctors and midwives lack the training to properly counsel on prenatal nutrition but still serve as a primary source of advice for pregnant women, and cultural beliefs may also conflict with national recommendations (2, 3). On top of this, current research provides differing assessments of the risk and benefit profiles of dietary components further confounding attempts for women to eat optimally during this time (4, 5).

This concern is particularly relevant for women with overweight or obesity [defined as a body mass index (BMI) ≥25 kg/m^2^ or ≥ 30 kg/m^2^, respectively], because excess body weight and body fat during pregnancy are associated with many health risks, including increased risk of cesarean section, premature delivery, excess birthweight, abnormal glucose and lipid metabolism, hypertensive disorders, postpartum hemorrhage and neonatal asphyxia (6–8). Hypertensive disorders, in particular, are associated with an increased risk of pulmonary edema, placental abruption, small-for-gestational age, and even perinatal death (9).

Not all prenatal nutrition recommendations are ambiguous. The importance of folate in early pregnancy to prevent neural tube defects and the detrimental effects of excessive alcohol consumption on offspring cognition have been well-documented, as has the importance of other micronutrients like choline and calcium, and omega-3 fatty acids (10–12). However, oftentimes, the evidence is less clear, for example when it comes to the macronutrient composition of the diet and individual food sources. Some studies support a dairy-rich diet for prevention of hypertensive disorders, while others have found no association (13–19). Relatedly, there is evidence that a diet rich in vegetable protein, but not animal protein, is inversely associated with blood pressure, though many of these effects do not persist once dietary confounders are considered and once results are adjusted for weight and BMI (20). It has also been hypothesized that the amino acid composition of different proteins, rather than the protein food source is the driving force behind the differing impacts on blood pressure, but here too, the results are mixed (21). Furthermore, some studies suggest diets high in fiber are optimal for blood pressure, but others provide mixed results (22). Ultimately though, few studies have examined the effect of the whole dietary pattern on blood pressure during pregnancy.

To address this fundamental gap in the literature, this secondary data analysis aimed to investigate the effect of consuming a diet rich in animal protein from dairy, seafood, and meat, with low glycemic index and load, versus consuming a diet following the Nordic Nutrition Recommendations, on blood pressure in pregnant females with overweight or obesity using data from the interventional study “An optimized programming of healthy children” (APPROACH) (23). We hypothesized that the additional dairy-derived protein and low glycemic index would be associated with beneficial changes in blood pressure regulation during pregnancy.

## Methods

### Design

The APPROACH study was a randomized controlled trial (RCT) conducted at Copenhagen University Hospital Herlev-Gentofte and the Department of Nutrition, Exercise and Sports (NEXS) at the University of Copenhagen, Denmark, from January 2014 to December 2017. This secondary data analysis includes data from the dietary intervention period of the study, until offspring birth. The females and their partners provided signed written informed consent before commencing participation. The study was approved by the Ethics Committee of the Capital Region of Denmark (H-3-2013-119) and registered at clinicaltrials.gov (NCT01894139).

### Subjects

The APPROACH study included 279 pregnant females (completers gave birth to 209 infants); this secondary data analysis includes all the participants from the original RCT. Recruitment procedures have been described in detail previously (23). Briefly, females were included from early second trimester (14 weeks and 3 days to 15 weeks and 4 days) and were included if they planned to give birth at Herlev hospital, had a pre-pregnancy BMI of 28–45 kg/m^2^, were older than 18 years of age, and were carrying a singleton pregnancy. Females were excluded for multiple pregnancies, dairy intolerance or allergy, weight loss greater than 10 kg over the last year, alcohol or drug abuse (defined as >14 units alcohol per week pre-pregnancy), or an eating disorder or other disease that might interfere with the intervention. Additionally, females with an oral glucose tolerance test diagnostic of gestational diabetes mellitus were excluded.

### Experimental procedures

The APPROACH study was a two-arm, single-blinded RCT, and has been described previously in detail (23). Briefly, the study was conducted in an outpatient setting in Denmark and the intervention lasted from early second trimester through birth. Prenatal females were randomized either to an *ad libitum* diet with high protein and low glycemic index (HPLGI), which was rich in dairy, seafood, and meat, or an *ad libitum* control diet with moderate protein and moderate glycemic index (MPMGI)—both aligned with the principles of the New Nordic Diet (23). The nutrient composition of the intervention and control diets is presented in Table 1.

**Table 1:** Nutrient composition of the prescribed experimental diets.

Component	HGLGI	MPMGI
Fat		
Total fat of which: Saturated and trans fatty acids Monounsaturated fatty acids (MUFA) Polyunsaturated fatty acids (PUFA)	30–32 E % ≤10 E % 10–15 E % 5–10 E %	30 E % ≤10 E % 10–15 E % 5–10 E %
Protein		
Total protein of which: From dairy From seafood	25–28 E % 8–10 E % ≥300 g/week	10–20 E % – 200 g/week
Carbohydrate		
Total carbohydrate Added sugar Fiber Glycemic Index	40-45 E % ≤10 E % 25–35 g/day ≤55	55–60 E % ≤10 E % 25–35 g/day 60
Micronutrients		
Calcium from diet Supplements	1,500 mg/d 1 supplement/day with 38 μg vitamin D, 200 μg folate, 40 mg iron, 2 μg vitamin B12, 650 mg/d omega 3 fatty acids [220 mg Docosahexaenoic acid (DHA)], 380 mg [Eicosapentaenoic acid (EPA)]	900 mg/d 1 supplement/day with 38 μg vitamin D, 200 μg folate, 40 mg iron, 2 μg vitamin B12, 650 mg/d omega 3 fatty acids (220 mg DHA, 380 mg EPA)

### Data collection

Habitual diet and adherence to the interventional diets was assessed through use of a Food Frequency Questionnaire (FFQ), based on a standardized and validated FFQ by Andersen et al. (24). The FFQ was administered at 15, 28, and 36 weeks of gestation, and was validated by two 24-h recalls at gestational weeks 21 and 32 (Supplementary Figure S1). Intakes of energy and individual nutrients were then calculated using DankostPro software (Kraftvaerk FoodTech, Denmark). Since the two experimental diets differed considerably in protein intake (25–28 vs. 15–18% of energy in the HPLGI and MPMGI diets, respectively), we assessed adherence to the dietary intervention objectively, by measuring urinary urea excretion (23).

Maternal height was measured to the nearest 0.5 cm at screening by using a wall mounted stadiometer (Seca, Germany). Pre-pregnancy weight was obtained either through self-report or through the participants’ medical records. Subsequent maternal body weights were measured to the nearest gram at screening, and at each of the 9 dietary counseling sessions throughout the intervention using a medical scale (Tanita, Illinois, United States) with the female being barefoot and in light clothing or undergarments. Gestational weight gain (GWG) was calculated as [*last reported weight before birth – pre-pregnancy weight*].

Blood pressure was measured by sphygmomanometer by the midwives at baseline (between gestational weeks 13–15), and then again at gestational week 21 (visit 3), between weeks 24 and 26 (visit 4), week 32 (visit 6), and between weeks 35 and 36 (visit 7) (Supplementary Figure S1). A calibrated blood pressure device approved by local medical authorities was used for each measurement, although the specific brand and model varied between and within participants. Per standard clinical practice, participants sat for at least 5 min prior to measurement, were instructed not to speak before and during measurement, and the cuff was placed on a bare arm (25). Blood pressure was measured once, unless, at the midwives’ discretion, an abnormal result prompted a second measurement. Mean arterial pressure was calculated as *diastolic blood pressure + (systolic blood pressure – diastolic blood pressure)/3* (26).

### Statistical analysis

Sample size calculations for the APPROACH study are based on the original primary outcome of the study, which was GWG in the maternal participants (23). There was no further sample size calculation done for this secondary analysis. Baseline characteristics of the participants were analyzed by independent t-test for continuous variables and are presented as mean ± standard deviation. For categorical variables, baseline characteristics were evaluated by chi-square test and presented as absolute counts and relative frequencies (Table 2). Dietary intake during the study was compared between groups by independent t-test and presented as mean ± standard deviation using an average from the 24-h recall data or from the FFQ data, depending on the method of evaluation for each dietary component reported (Table 3). The effect of the diet on blood pressure was evaluated using a linear mixed model which included time and group (intervention/control) as fixed factors, a group-by-time interaction, and subject ID as random intercept. This type of model was selected as it allows for missing values and is most suitable when there may be correlations between repeated measures. Systolic, diastolic and mean arterial pressures were analyzed in separate linear mixed models. A model with adjustment for GWG was also constructed. Frequency of blood pressure-related complications between the two groups was evaluated by a chi-square test. The data were visually assessed for statistical assumptions, and there were no clear violations in normality, linearity, and homoscedasticity in the residual plots. A two-sided significance of *p* < 0.05 was used, and the analyses were carried out in SPSS Statistics for Mac version 28 (SPSS inc. Chicago, Ill, United States).

**Table 2:** Characteristics of participants at baseline.

	HPLGI	MPMGI	All	HPLGI vs MPMGI
Age (years)^1^	30.7 ± 4.6	30.0 ± 4.9	30.4 ± 4.8	*p* = 0.239
Race—white (%)/non-white (%)^1^	133 (94.3%)/8 (5.7%)	127 (92.0%)/11 (8%)	260 (93.2%)/19 (6.8%)	*p* = 0.580
Education (level, from 0 to 3)^1,2^	0.5 ± 0.5	0.5 ± 0.5	0.5 ± 0.5	*p* = 0.060
BMI (kg/m^2^)^1,4^	34.0 ± 3.8	34.3 ± 3.8	34.2 ± 3.8	*p* = 0.487
Systolic blood pressure^4,5^ (mmHg)	120.7 ± 10.3	121.8 ± 11.2	121.2 ± 10.7	*p* = 0.447
Diastolic blood pressure (mmHg)^4,5^	75.4 ± 9.6	76.9 ± 8.2	76.2 ± 8.9	*p* = 0.232
Smoking (cigarettes/wk)^6^	1.9 ± 8.4	3.1 ± 15.4	2.5 ± 12.4	*p* = 0.435
Alcohol^7^ (drinks/mo)	0.1 ± 0.4	0.1 ± 0.6	0.1 ± 0.5	*p* = 0.561
Parity-multiparous (%)/nulliparous (%)	67 (48.2%)/72 (51.8%)	64 (48.1%)/69 (51.9%)	131 (48.2%)/141 (51.8%)	*p* = 0.989
Calcium intake^8^ (mg)	1129.8 ± 549.2	1089.6 ± 599.7	1110.1 ± 573.8	*p* = 0.564

^1^n intervention = 141, n control = 138. ^2^Education rated on a scale of 0–3 where 0 is no education/unskilled and 3 at least 5 years of higher education. ^3^n intervention = 131, n control = 138. ^4^Measurement taken pre-baseline, at screening. ^5^n intervention = 96, n control = 97. ^6^n intervention = 136, n control = 133. ^7^n intervention = 137, n control = 13. ^8^n intervention = 139, n control = 134.

**Table 3:** Average dietary intake during intervention.

Component	HPLGI	MPMGI	HPLGI vs. MPMGI
Total fat (E %)^1,2,3,4^ Saturated fat (E %)^2,3^ MUFA (E %)^2,3^ PUFA (E %)^2,3^	32.3 ± 6.1 10.6 ± 3.4 11.6 ± 2.9 5.3 ± 1.7	30.1 ± 6.2 10.2 ± 3.5 10.3 ± 2.9 5.3 ± 1.5	*p* = 0.007 *p* = 0.329 *p* < 0.001 *p* = 0.913
Total protein (E %)^2,3^ Protein from dairy (E %)^2,3^ Protein from seafood (E %)^5,6^	25.1 ± 4.7 10.0 ± 3.9 13.6 ± 10.4	18.1 ± 3.3 4.9 ± 4.3 10.1 ± 10.2	*p* < 0.001 *p* < 0.001 *p* = 0.011
Total carbohydrate (E%)^2,3^ Total sugar (E %)^2,3^ Fiber (g/100 kcal/d)^3,7^ Glycemic index^8,9^	42.7 ± 6.5 3.0 ± 4.2 14.2 ± 6.0 49.5 ± 4.8	51.8 ± 6.4 4.2 ± 4.2 17.3 ± 7.2 54.1 ± 3.7	*p* < 0.001 *p* = 0.022 *p* < 0.001 *p* < 0.001
Calcium (mg/100 kcal/d)^2,9^	882 ± 400	654 ± 358	*p* < 0.001
Potassium (g/d)	2.7		*–*
Magnesium (mg/d)	262		*–*
Sodium (mg/d)	2489 ± 961	2277 ± 972	*p* = 0.093
Meat (g/d)	158.1 ± 152.9	101.1 ± 98.8	*p* < 0.001

^1^%, percentage of total energy consumption. ^2^Presented as percent of total energy intake from an average of two 24-hr recalls. ^3^*n* = 120 (intervention), *n* = 117(control). ^4^Total fat calculated as the sum of polyunsaturated fatty acids, monounsaturated fatty acids, saturated fatty acids, n-3 fatty acids and n-6 fatty acids, in the Frida food database. ^5^*n* = 120 (intervention), *n* = 115 (control). ^6^Presented as percent of total energy intake from an average of three FFQs. ^7^Presented in grams per 1,000 kcal/day as an average from two 24-h recalls. ^8^*n* = 140 (intervention), *n* = 134 (control). ^9^Calculated from an average of three FFQs.

## Results

The whole sample included 279 pregnant females, with 141 of them assigned to the intervention group and 138 to the control group (Table 2). Participants were 30.4 ± 4.8 years old, with a pre-pregnancy BMI of 34.2 ± 3.8 kg/m^2^, and most of them were white (260 of 279). There were no significant differences in age, race, BMI, education level, blood pressure, weight, smoking status, alcohol use, or parity between the groups at baseline (Table 2). Over the course of the intervention, 70 of the 279 randomized females (25%) dropped out of the study, with no difference between groups. The females in the intervention group had an average GWG of 6.8 ± 1.3 kg, which was 1.7 kg less (*p* < 0.05) than those in the control group who gained an average of 8.5 ± 1.3 kg; both groups had a GWG that fell within the Institute of Medicine guidelines (23). A total of 100 (35.8%) of the participants experienced a blood pressure value above normal limits (120/80 mm Hg) at some point during pregnancy, though this did not differ by group (*p* = 0.594). Average vitamin D levels during pregnancy were 67 nmoL/L.

### Dietary intake

As per allocation, females in the intervention group increased their consumption of dairy protein to 10% of total energy intake, whereas those in the control group continued to consume roughly 5% of total energy as dairy protein, with the majority of dairy being low-sodium high-protein yogurt and skyr (*p* < 0.001) (Table 3). The HPLGI group consumed roughly 3.5% more of their daily energy intake from seafood protein than the MPMGI group, but polyunsaturated fatty acid intake was not different between groups (10.4 g/d in the MPMGI group, 10.5 g/d in the HPLGI group). The intervention and control groups consumed an average of 1,415 and 1,106 mg of calcium per day, respectively, from dairy, supplements, and other food sources (Table 3). On average, the participants daily consumed 2.7 g of potassium and 262 mg of magnesium, and approximately 2.4 g of sodium (Table 3). Sodium intakes were similar between groups. As reported previously, dietary adherence, assessed by urinary urea excretion, was high in both intervention groups and did not differ between them (22).

### Blood pressure

**Figure 1:**
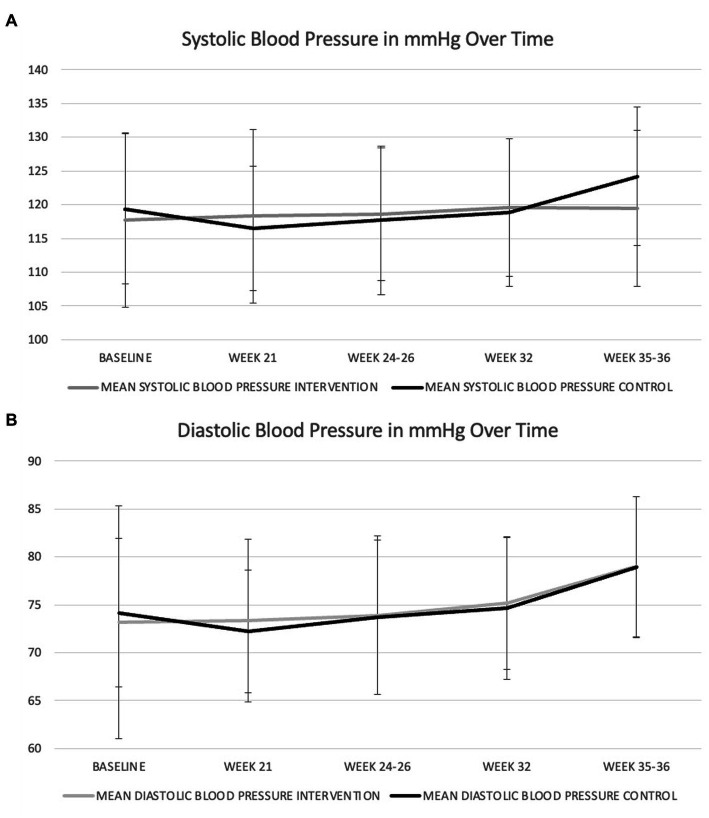
Changes in systolic **(A)** and diastolic **(B)** blood pressure during pregnancy in women consuming the dairy-rich diet and those consuming the average Danish diet. Data are means and standard deviations.

In the unadjusted analysis, there was a significant effect of time with systolic blood pressure increasing during pregnancy (Figure 1A), but there was no significant effect of diet on systolic blood pressure (*p* = 0.790). There was also no significant group-by-time interaction (Table 4). In the GWG-adjusted analysis (the only known potential confounder that differed between groups), the effect of group remained non-significant (*p* = 0.760) (Table 4). Similar results were obtained for diastolic blood pressure (Figure 1B and Table 5) and mean arterial pressure (Table 6).

**Table 4:** Effect of animal protein-rich diet on systolic blood pressure during pregnancy*.

	Parameter	Estimates	Significance
Unadjusted model	Constant	118.1 (115.9, 120.3)	*p* < 0.001
	Group (ref = 0; control)	−0.3 (−2.6 – 2.0)	*p* = 0.790
	Time (overall)	–	*p* < 0.001
	−1	–	*–*
	−2	0.2 (−2.3, 2.6)	*p* = 0.895
	−3	0.5 (−1.4, 2.5)	*p* = 0.593
	−4	1.5 (−0.4, 3.4)	*p* = 0.131
	−5	4.5 (2.3, 6.7)	*p* < 0.001
	**Parameter**	**Estimates**	**Significance**
Adjusted model	Constant	123.1 (120.3, 125.8)	*p* < 0.001
	Group (ref = 0; control)	−0.4 (−3.0, 2.2)	*p* = 0.760
	Time	–	*p* < 0.001
	Gestational weight gain	0.014 (−0.2, 0.2)	*p* = 0.902

*Includes data from *n* = 214 pregnant females.

**Table 5:** Effect of animal protein-rich diet on diastolic blood pressure during pregnancy*.

	Parameter	Estimates	Significance
Unadjusted model	Constant	73.6 (71.9, 75.3)	*p* < 0.001
	Group (ref = 0; control)	−0.3 (−1.9, 1.3)	*p* = 0.720
	Time (overall)	−	*p* < 0.001
	−1	–	–
	−2	−0.8 (−2.6, 1.0)	*p* = 0.372
	−3	0.2 (−1.5, 1.2)	*p* = 0.819
	−4	1.4 (−0.2, −3.1)	*p* = 0.080
	−5	5.4 (3.7, 7.1)	*p* < 0.001
	**Parameter**	**Estimates**	**Significance**
Adjusted model	Constant	73.8 (71.5, 76.0)	*p* < 0.001
	Group (ref = 0; control)	0.3 (−1.5, 2.0)	*p* = 0.767
	Time	–	*p* < 0.001
	Gestational weight gain	−0.1 (−0.3, 0.0)	*p* = 0.131

*Includes data from *n* = 214 pregnant females.

**Table 6:** Effect of animal protein-rich diet on mean arterial pressure during pregnancy*.

	Parameter	Estimates	Significance
Unadjusted model	Constant	88.4 (86.8, 90.0)	*p* < 0.001
	Group (ref = 0; control)	−0.4 (−2.0, 1.3)	*p* = 0.651
	Time (overall)	−	*p* < 0.001
	−1	–	*–*
	−2	−0.4 (−2.1, 1.2)	*p* = 0.602
	−3	0.3 (−2.1, −1.8)	*p* = 0.642
	−4	1.5 (0.1, 2.9)	*p* = 0.038
	−5	5.1 (3.7,6.7)	*p* < 0.001
	**Parameter**	**Estimates**	**Significance**
Adjusted model	Constant	88.5 (86.3, 90.7)	*p* < 0.001
	Group (ref = 0; control)	−0.3 (−2.1, 1.5)	*p* = 0.775
	Time	−	*p* < 0.001
	Gestational weight gain	−0.1 (−0.2, 0.1)	*p* = 0.321

*Includes data from *n* = 214 pregnant females.

### Blood pressure related birth and pregnancy outcomes

There were no differences between groups in the prevalence of premature birth, preeclampsia, or hypertension. There was, however, a significant difference in the prevalence of planned and total cesarean sections between groups, though there was no difference in acute cesarean sections (Table 7). There was no difference between groups in regard to previous complicated pregnancies, an important medical history which can lead to subsequent planned cesarean section. When pregnancy and birth complications were evaluated as a composite score, there were more complications in the control group than in the experimental group (*p* = 0.008).

**Table 7:** Incidence of blood pressure-associated events between groups.

	HPLGI	MPMGI	Significance
Hypertension—yes (%)/no (%)	0 (0.0%)/138 (100.0%)	1 (0.7%)/141 (99.3%)	*p* = 0.322
Premature Birth—yes (%)/no (%)	3 (2.8%)/103 (97.2%)	7 (6.3%)/104 (93.7%)	*p* = 0.200
Preeclampsia—yes (%)/no (%)	1 (1.0%)/97 (99.0%)	1 (1.0%)/101 (99.0%)	*p* = 0.977
Unplanned cesarean section—yes (%)/no (%)	13 (11.1%)/104 (88.9%)	20 (16.1%)/104 (83.9%)	*p* = 0.184
Planned cesarean section—yes (%)/no (%)	3 (2.8%)/104 (97.2%)	10 (8.8%)/104 (91.2%)	*p* = 0.045
Total cesarean sections— yes (%)/no (%)	16 (13.3%)/104 (86.7%)	30 (22.4%)/104 (77.6%)	*p* = 0.019
Composite complication score—yes (%)/no (%)	20 (14.5%)/118 (85.5%)	39 (27.7%)/103 (72.3%)	*p* = 0.008
Gestational diabetes—yes (%)/no (%)	12 (8.5%)/129 (91.5%)	6 (4.3%)/132 (95.7%)	*p* = 0.157

Data are absolute frequencies and have been compared between groups with chi-square test. Composite Complication Score is a summation of all above blood-pressure associated events.

## Discussion

### Main findings

Blood pressure increased during pregnancy in our participants, particularly during the last trimester, but it was largely unaffected by the experimental diet consumed during pregnancy, or the amount of GWG, and did not enter the hypertensive range for most participants. Although 40% of the participants experienced a blood pressure value above normal limits (120/80 mm Hg) at some point during pregnancy, there were few hypertension-related adverse pregnancy outcomes in either group, and diet did not seem to be significantly associated with their prevalence, except in the case of planned cesarean section. These results indicate that a diet rich in dairy, seafood, and meat, which follows the national recommendations, is not associated with unfavorable changes in blood pressure during pregnancy or adverse maternal outcomes.

### Interpretation

Obesity is associated with higher serum parathyroid hormone (PTH) levels derived from increased vitamin D deposition in adipose tissue. This reduces systemic vitamin D bioavailability and subsequently impairs the negative feedback mechanism between vitamin D and PTH (19, 20). The obesity-driven increase in PTH could give rise to an increase in intracellular calcium and a proportional decrease in extracellular calcium—preventing the purported action of calcium on blood pressure and increasing vascular resistance (21). Furthermore, active calcium transport from the intestine depends on the binding of calcitriol [1,25(OH)_2_D3] to its vitamin D receptor and is diminished when vitamin D status is impaired (22). Accordingly, the physiological effects of dairy-derived micronutrients, which were consumed to greater amounts in the experimental group, may be attenuated in subjects with obesity.

The United States Endocrine Society recommends a 25-hydroxyvitamin D level of around 75 nmoL/L as normal during pregnancy, though some literature suggests a level of 100 nmoL/L for optimal maternal and offspring outcomes (27, 28). However, in Denmark, sufficiency is set much lower—at only 50 nmoL/L (29). Despite supplementation with 38 μg/d vitamin D throughout the study, well-above the recommended intake for Denmark, females in the APPROACH trial had average vitamin D levels of 67 nmoL/L—below the US Endocrine Society recommendations (29). At this level, though, it is possible that the females had suboptimal calcium absorption contributing to the lack of an effect of additional dairy-derived calcium on blood pressure and contributing to nearly half of them having an elevated blood pressure measurement at least once during the study.

Another potential mechanism involves obesity and the inhibition of magnesium and potassium’s role in nitric oxide production and endothelial relaxation and vasodilation (30). In obesity, an increased abundance of pro-inflammatory cytokines is associated with decreased nitric oxide and increased endothelial vasoconstriction—possibly countering the dairy-derived magnesium nitric oxide production, and both magnesium and potassium’s role in endothelial relaxation (31–33). Additionally, normal pregnancy is associated with increased oxidative stress, which is exacerbated in obesity (34). If persistent, the increased reactive oxygen species formation has the potential to inactivate nitric oxide (35, 36). This, too, can increase endothelial vasoconstriction and counteract the beneficial effects of magnesium and potassium on blood pressure, thereby contributing to a lack of an effect of the additional dairy consumption on blood pressure (32, 33).

Despite the high consumption of dairy foods by the participants in the experimental group, intakes of some of the blood pressure-associated micronutrients found in dairy was—in both groups—below the Nordic Nutrition Recommendations (i.e., potassium and magnesium) (29). Also, although both groups of women consumed adequate amounts of calcium; and clinical trials observing a significant reduction in blood pressure with 500–2,000 mg/d calcium supplementation did so at a higher supplemental dosage than APPROACH intakes (29, 37, 38). It is therefore conceivable that intake of these micronutrients was not sufficient to cause a beneficial effect on blood pressure in our participants, particularly given the added physiological challenges of pregnancy and obesity. Though statistically significant, the differences between groups in fish intake, a food typically rich in anti-inflammatory omega-3 fatty acids, may not have been large enough to induce clinically meaningful changes in any of the reported outcomes. This could also explain a potentially similar lack of effect from differences in glycemic index and fiber. However, given the conflicting results on the impact of glycemic index and fiber on blood pressure, it is also possible that this aspect of the prenatal diet does not play a critical role in the regulation of blood pressure during pregnancy. Most relevant studies have been observational, and none has thoroughly explored potential mechanisms (22).

The pregnancies in the present study were largely uncomplicated, particularly in regard to outcomes that might have been influenced by obesity and blood pressure. The consumption of similar amounts of fat, but with larger differences in carbohydrates, glycemic index, and protein, had no impact on the blood pressure responses during pregnancy in this at-risk group of women. Furthermore, the women consumed relatively high amounts of saturated fat, above the recommendations of <10% of daily intake, and close to the upper limit of the recommendation for sodium (2.4 g/d) with no adverse impact on their health during pregnancy, or on the health of their offspring (29). Both diets encouraged women to consume a variety of foods that fit within the guidelines of their assigned dietary intake pattern—those in the experimental group adhered to an *ad libitum* diet with high protein and low glycemic index, which was rich in dairy, seafood, and meat, and those in the control group to an *ad libitum* diet with moderate protein and moderate glycemic index.

### Strengths and limitations

The study had several strengths. The combination of several FFQ and 24-HR has been shown to increase precision of food intake estimates—crucial in nutrition and diet studies (39). Furthermore, following and obtaining data on the women from early second trimester until just prior to birth allowed us more accurately understand the trajectory of blood pressure and its interplay with diet throughout pregnancy. Furthermore, this study is the first of its kind in pregnant women with obesity.

However, the study also had several limitations. Blood pressure was not recorded after gestational week 37 and until birth, when a potential spike in blood pressure might have occurred. The relatively small sample size might have increased the likelihood of type 2 error, particularly regarding detecting blood-pressure related pregnancy complications. Lastly, a challenge of secondary data analysis is the inability to control the data collection process retrospectively. Though there is a standard for blood pressure measurement in Denmark, the protocol had no standard operating procedure for blood pressure, and there was no case report form confirming details which may alter blood pressure reading, including type of cuff used (manual or digital), placement of cuff over clothing, talking during the measurement, body positioning during measurement, and amount of time resting beforehand (40, 41). There is also no standard of care requirement for repeated measurements of blood pressure by the midwives involved in the treatment of the participants during pregnancy. Similarly, pre-pregnancy body weight was obtained either from the electronic medical record or self-report, increasing potential for reporting bias. Furthermore, the dietary intake might have been under-reported, as is common among people with overweight or obesity (42). However, the assessment of urea nitrogen reflected a high degree of adherence to, and a clear separation between, the experimental diets. Lastly, the APPROACH intervention was conducted in Copenhagen, Denmark, in a relatively homogeneous population, so the possibility that the results are not generalizable to other populations cannot be discounted.

## Conclusion

Ultimately, the two diet patterns made little difference on blood pressure physiology, contradicting the 2021 American Heart Association’s guidelines for cardiovascular health which provide counsel on appropriate protein types for optimal health (43). These results provide early evidence that a varied and healthy dietary pattern contributes to healthy and successful pregnancies, regardless of its macronutrient composition and food sources, and the accompanying small variation in GWG. While further studies powered for blood pressure as the primary outcome are needed to confirm these novel findings, this secondary data analysis of the APPROACH trial addresses a critical gap in the literature by examining the effect of whole dietary patterns on blood pressure during pregnancy and puts forth some preliminary evidence which may aid in dispelling some of the myth of the “perfect” diet for pregnancy.

## Data availability statement

The data analyzed in this study is subject to the following licenses/restrictions: data may be requested from the authors of the original paper. Requests to access these datasets should be directed to nge@cslt.dk.

## Ethics statement

The studies involving humans were approved by Ethics Committee of the Capital Region of Denmark (H-3-2013-119). The studies were conducted in accordance with the local legislation and institutional requirements. Written informed consent for participation in this study was provided by the participants’ legal guardians/next of kin.

## Author contributions

EL: Conceptualization, Data curation, Formal analysis, Investigation, Methodology, Project administration, Software, Writing – original draft, Writing – review & editing. FM: Data curation, Methodology, Software, Supervision, Writing – review & editing. HZ: Writing – review & editing. JS: Writing – review & editing. AA: Writing – review & editing. NG: Conceptualization, Data curation, Investigation, Methodology, Supervision, Writing – review & editing.

## Supplementary material

SUPPLEMENTARY FIGURE S1 Timeline of relevant study events.
