# Supplementary figures and images for: A high protein low glycemic index diet has no adverse effect on blood pressure in pregnant women with overweight or obesity: a secondary data analysis of a randomized clinical trial
Source: Front Nutr. 2023 Nov 21;10:1289395. doi: 10.3389/fnut.2023.1289395 (PMC10702593; doi:10.3389/fnut.2023.1289395)

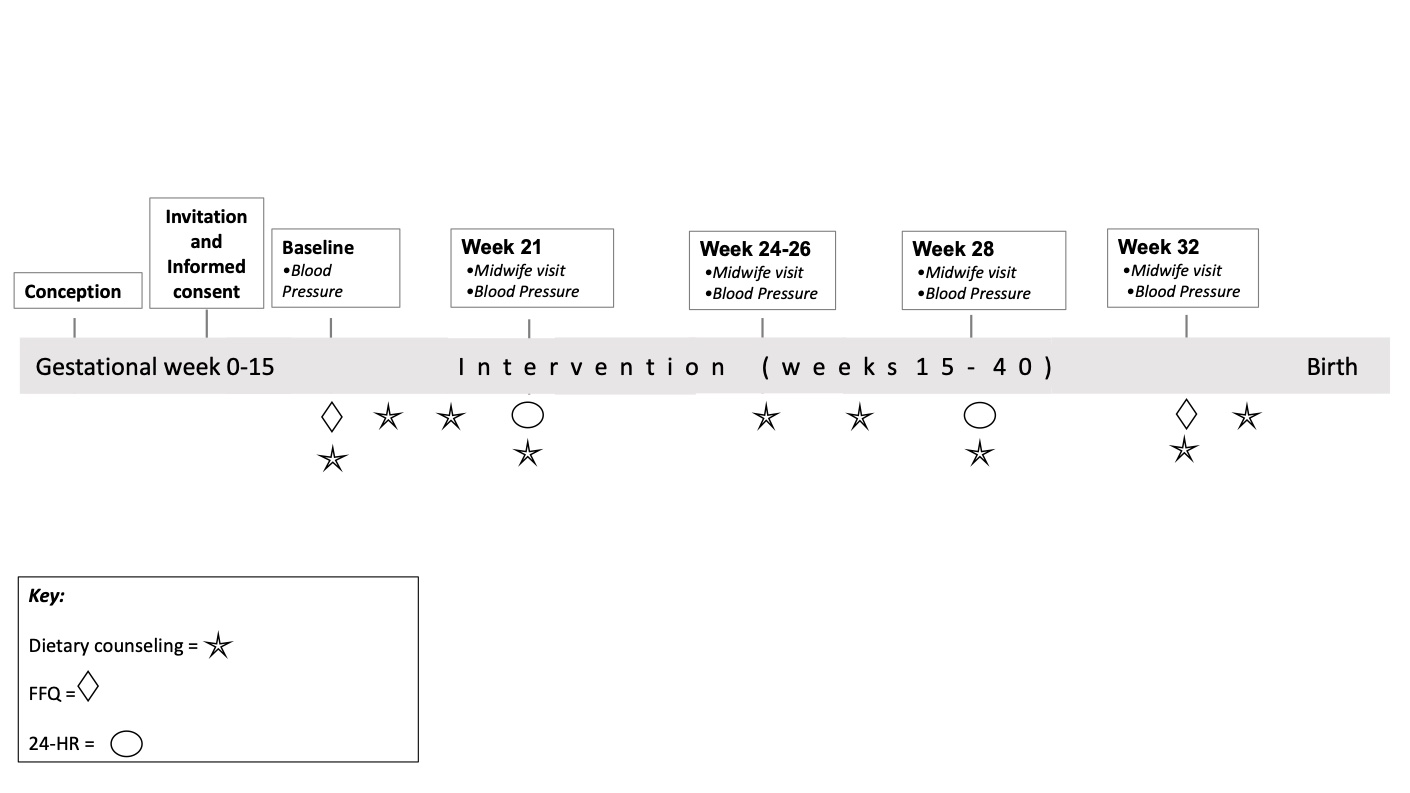

Supplement: SUPPLEMENTARY FIGURE S1 — Timeline of relevant study events. [file Image_1.jpg]
